# The Role of Radiation Therapy as an Adjuvant Treatment in Nodal Metastasis of Malignant Chondroid Syringoma

**DOI:** 10.7759/cureus.11360

**Published:** 2020-11-06

**Authors:** Sergio L Favareto, Antonio Cassio A Pellizzon, Clóvis A Lopes Pinto, Eduardo Bertolli, Douglas G Castro

**Affiliations:** 1 Radiation Oncology, Antônio Cândido de Camargo Cancer Center, São Paulo, BRA; 2 Anatomic Pathology, Antônio Cândido de Camargo Cancer Center, São Paulo, BRA; 3 Skin Cancer Department, Antônio Cândido de Camargo Cancer Center, São Paulo, BRA

**Keywords:** malignant chondroid syringoma, nodal metastasis, radiotherapy

## Abstract

Malignant chondroid syringomas (MCSs) are extremely rare and aggressive skin tumors, and wide surgical excision is the main treatment. They can progress with local recurrence and nodal and distant metastasis. The role of radiotherapy is uncertain, but may enhance local control after surgical approach. We report a case of a 60-year-old female with this disease that, four years after surgical resection, presented with nodal metastasis and was managed with surgery and adjuvant radiotherapy.

## Introduction

Chondroid syringomas (CSs) are rare benign tumors originated from sweat gland and ectopic salivary glands. They are usually asymptomatic, indolent and occur usually in the head and neck [1,2]. Malignant chondroid syringomas (MCSs) are extremely rare and, on the other hand, are much more aggressive than the benign form and occur usually on extremities and trunk. They can recur locally, besides nodal and distant metastasis. Surgical resection is the standard of care when there is only local disease, but there is no specific treatment on metastasis cases [3-5]. The use of radiation therapy and its response is unknown for MCS.

Herein, we present a case of a 60-year-old female diagnosed with MCS on the lateral aspect of the left knee, treated with surgical resection. She was found to have inguinal and iliac nodal metastasis four years later and was successfully managed with radiation therapy.

## Case presentation

**Figure 1:**
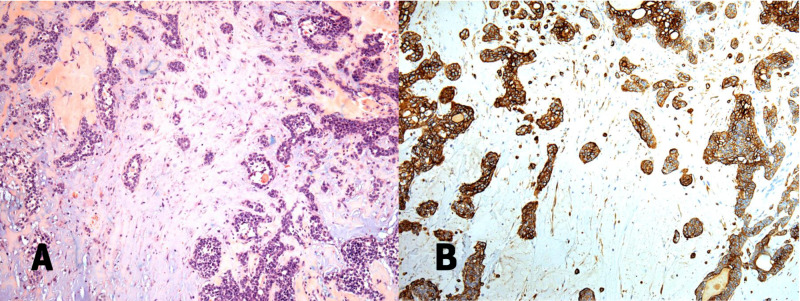
(A) Photomicroscopy in hematoxylin and eosin (100x), showing a neoplasm characterized by epithelial structures of glandular and trabecular patterns in the middle of the myxoid and chondroid matrix; (B) Immunohistochemistry photomicroscopy of a CK7 antibody (100x) showing cytokeratin 7 with diffuse expression in the glandular component.

**Figure 2:**
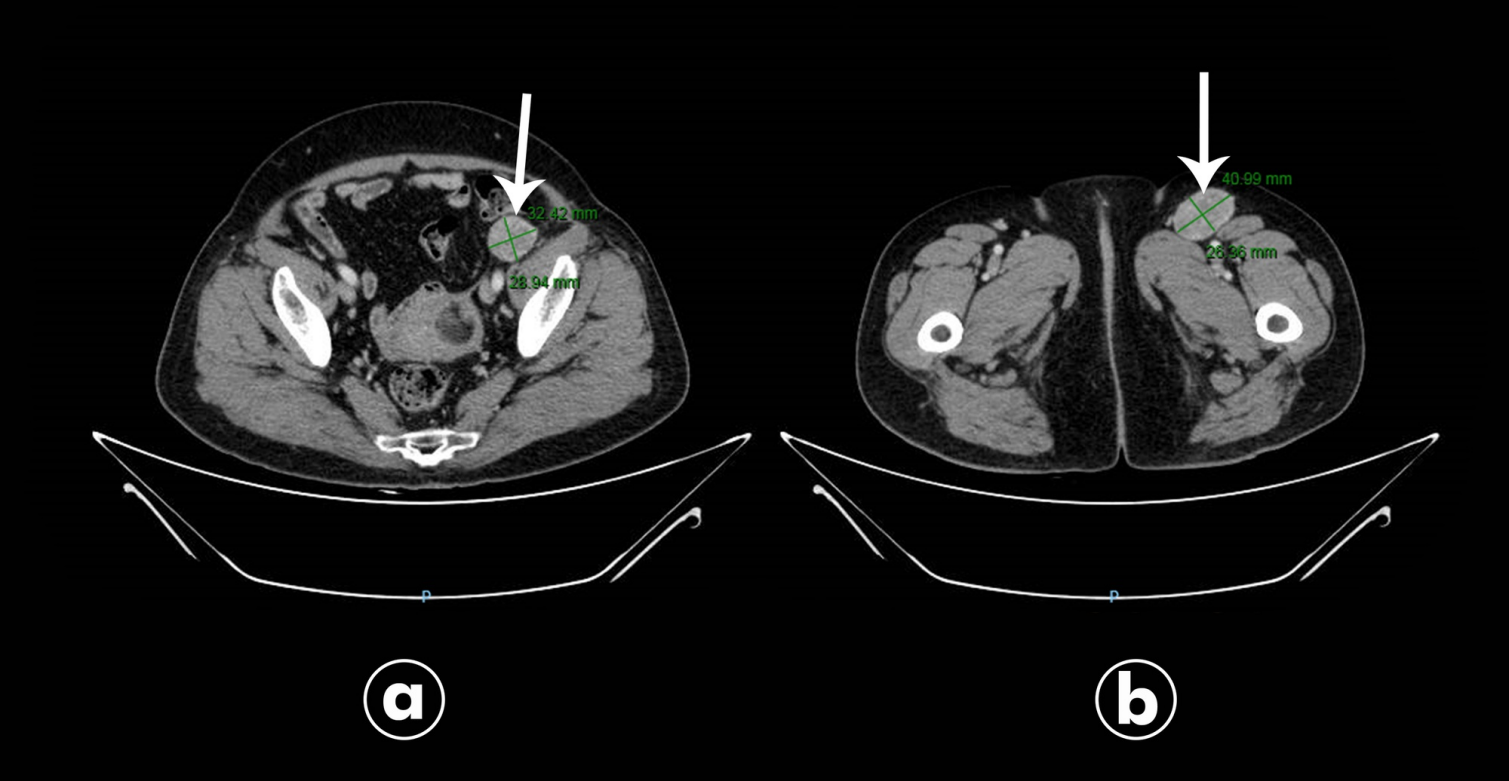
CT of the pelvis with heterogenous lymph nodes enlargement: (a) in the left external iliac chain, and (b) in the left inguinal region.

**Figure 3:**
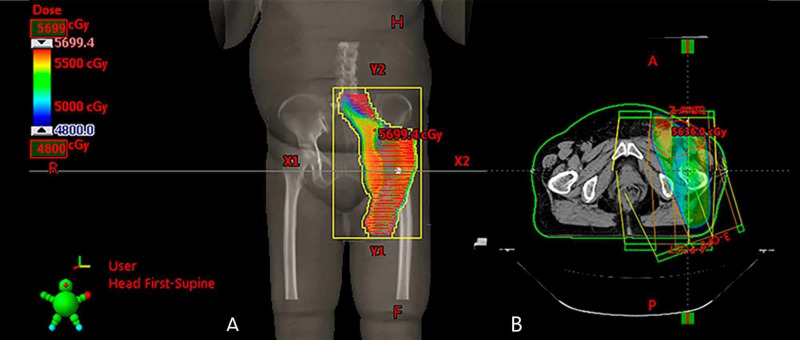
(A) Beam's eye-view of the anterior field; (B) Treatment planning with 3D technic and dose distribution between 4800 and 5699.4 cGy in the isocenter.

A 60-year-old female presented with a nodular lesion in the lateral side of the left knee, measuring 2 cm, painful on palpation, that appeared two years before. She underwent a resection in 2013 and the histology confirmed MCS, with positive margins (Figure 1). The patient then underwent a local excision after 45 days and the histology found a residual MSC with free margins. Four years later, in November 2017, she presented with an inguinal node, and fine-needle aspiration was performed and confirmed positive cytology for malignancy, compatible with metastasis of basaloid neoplasm. Immunohistochemical staining suggested metastasis of MSC. A computed tomography (CT) of the pelvis showed heterogeneous lymph nodes enlargement, with intense enhancement of contrast, the 2 biggest in the left inguinal region, measuring 42 x 27 mm, and in the left external iliac chain, measuring 31 x 27 mm (Figure 2). There was no evidence of other sites of disease. After a multidisciplinary discussion, she underwent inguinal and iliac lymphadenectomy in February 2018, with four of nine lymph nodes harvested with metastasis of basaloid neoplasm in the left inguinal region, with extracapsular spread (7.5 cm); two of four lymph nodes in the pelvic region, without extracapsular spread (1.0 mm); and one of one lymph node in the left external iliac region, with extracapsular spread (5.2 cm). Due to the number of involved lymph nodes and the presence of extracapsular spread, adjuvant three-dimensional conformal radiation therapy in the left inguinal and iliac region was performed, with a total dose of 48Gy in 20 fractions of 240cGy (Figure 3), until May 2018. It was not performed any systemic therapy. The patient developed mild lymphoedema on her left leg after the treatment, which was controlled with clinical measures. She is being followed with periodic exams, including pelvic and thoracic CTs, and shows no sign of disease until March 2020.

## Discussion

CSs are rare mixed skin tumors, composed of both epithelial and stromal components, originated from cells of sweat gland and ectopic salivary gland [1]. They were first described in 1961, by Hirsch and Helwig [2], and are usually located on the head and neck, asymptomatic and presenting a slow-growing, classified as a benign disease.

MCS is the malignant form of this illness, with less than 50 cases reported. It is usually localized on extremities and trunk, grows quickly and invasively, occurs predominantly in females (1.7:1), has no definite age predilection, with median age of 49 years, ranging from 13 to 83, and has no evidence of a specific risk factor [3-5]. Most of the time, MCS appears de novo; however, a CS, after many years of evolution, can undergo malignant transformation and become metastatic.

The histological features that are suggestive of this pathology, differentiating from the benign form, include nuclear pleomorphism, cytologic atypia, increased mitotic activity, focal necrosis and lymphatic or vascular invasion [6]. Immunochemistry shows a positivity for epithelial markers (cytokeratin), epithelial structures, vimentin, PS100, actin and calponin for both smooth muscle and myoepithelial cells [3]. Among differential diagnosis, mucinous carcinoma and myxoid chondrosarcoma are included.

Adequate wide surgical excision is the main treatment for MCS. From cases reported so far, 50% have local recurrences. Nodal metastasis was observed in 39% of the cases, while distant metastases (lung, bone and brain) were observed in 36% of them. 27% of patients died from this disease within nine weeks following surgery; one patient survived 13 years after diagnosis [1,4].

There are only three cases report of the use of radiation therapy on MSC. Schvili and Rothem [7] reported this approach as an adjuvant treatment after nodal metastasis resection, but not the techniques, volume and dose that was used. Hong et al. [8] referred an adjuvant radiotherapy after the resection of a local recurrence, using 5000 cGy in 25 fractions on the tumor site with 2 cm margins, with no evidence of recurrence or metastatic disease after 27 months of follow-up. Watarai et al presented a case of MCS recurrence treated with surgery, radiation therapy and chemotherapy combination, with no clinical progression after 18 months of follow-up. Dose of radiation was not mentioned [9]. 

In this reported case, as there is no specific treatment for MCS lymph metastasis, we based the treatment on the intergroup multicenter randomized trial (ANZMTG 01.02/TROG 02.01), which includes adjuvant radiotherapy for patients with melanoma after lymphadenectomy at high risk of recurrence (according to the size of involved nodes and the presence of extracapsular extension) [10].

A total dose of 4800 cGy in 20 fractions of 240 cGy with three-dimensional conformal radiation therapy was performed on the left inguinal and iliac region. The only complication the patient presented was mild lymphoedema, that is expected after this sort of treatment (surgery + radiotherapy). No sign of residual disease was found after one year and three months of follow-up and no systemic therapy was used.

## Conclusions

MCS is an extremely rare condition and its management remains controversial. It is more aggressive than the benign form and, as there are only a few cases reported and each one has differences among them, systematic approach lacks. Complete resection is the main treatment and the role of radiotherapy on this disease remains uncertain, but it seems to be a reasonable option as adjuvant treatment for optimizing local control after nodal metastasis resection. Clinicians, including primary care physicians and dermatologists, should be aware of the existence of this entity and try to biopsy any suspicious lesion to rule out possible underlying malignancy.
